# Hepatitis C Vaccination: Where We Are and Where We Need to Be

**DOI:** 10.3390/pathogens10121619

**Published:** 2021-12-14

**Authors:** Vignan Manne, John Ryan, Jonathan Wong, Gayatri Vengayil, Syed Abdul Basit, Robert G. Gish

**Affiliations:** 1 HCA Healthcare Graduate Medical Education, Las Vegas, NV 89148, USA; Vignan.Manne@hcahealthcare.com (V.M.); Jonathan.Wong@hcahealthcare.com (J.W.); Gayatri.Vengayil@hcahealthcare.com (G.V.); 2 Comprehensive Digestive Institute of Nevada, Las Vegas, NV 89148, USA; ryan@nevadagastro.com (J.R.); sab@nevadagastro.com (S.A.B.); 3 Liver Transplant Clinic, Loma Linda University, Loma Linda, CA 92350, USA

**Keywords:** hepatitis C virus, vaccination, chronic liver disease, direct acting anti-virals (DAAs)

## Abstract

The hepatitis C virus (HCV) is a common cause of chronic liver disease and liver cancer worldwide. Despite advances in curative therapies for HCV, the incidence of new infections is not decreasing at the expected rate to hit the World Health Organization (WHO) target for the elimination of HCV by 2030. In fact, there are still more new cases of infection in the United States and worldwide than are being cured. The reasons for the rise in new cases include poor access to care and the opioid epidemic. The clinical burden of HCV requires a multimodal approach to eradicating the infection. Vaccination would be an excellent tool to prevent incidence of new infections; however, the genetic diversity of HCV and its ability to generate quasispecies within an infected host make creating a broadly reactive vaccine difficult. Multiple vaccine candidates have been identified, but to date, there has not been a target that has led to a broadly reactive vaccine, though several of the candidates are promising. Additionally, the virus is very difficult to culture and testing candidates in humans or chimpanzees is ethically challenging. Despite the multiple barriers to creating a vaccine, vaccination still represents an important tool in the fight against HCV.

## 1. Introduction

The hepatitis C virus (HCV) is one of the most common causes of chronic liver disease, cirrhosis, liver transplant, and liver cancer worldwide, and is also a systemic infection with effects on many organ systems and quality of life. Acute infection with HCV generally becomes a chronic condition, with up to 75% of patients remaining HCV ribonucleic acid (RNA) positive, and thus often leads to liver disease-related morbidity and mortality [1]. Since the 1980s, the prevalence of new HCV infections has decreased in some countries, but due to a multitude of factors, the incidence is rising in other countries and blunting the change in prevalence. With the advent of the direct-acting antiviral (DAAs) therapies, the treatment and cure of chronic HCV has significantly improved [1].

Despite improvements in screening, treatment, and linkage to care for at-risk populations, a majority of people with chronic hepatitis C (CHC) have not been tested or treated in most countries [2]; the exceptions are global leaders such as Egypt and Georgia where screening and linkage to care is at very high levels. Several theories have been postulated as to why this is the case, but regardless of the reasons, it is abundantly clear that preventive strategies would augment our efforts to manage the burden caused by HCV. A vaccine for HCV would be an ideal preventive solution to add to the arsenal; however, due to the genetic diversity of the virus and an incomplete understanding of the body’s immune response to the virus, vaccine development continues to be in experimental stages. This article reviews the latest information surrounding HCV vaccine development.

## 2. Clinical Need for Hepatitis C Virus Vaccine

Worldwide, roughly 100 million people have been infected with HCV (i.e., anti-HCV positive) at an annual incidence of 3–4 million/year, along with an estimated 71 million people thought to have CHC (i.e., HCV RNA positive) [1,3]. The prevalence of HCV varies greatly from region to region, with the eastern Mediterranean region having the highest prevalence, at 2.3%, and the western Pacific region having the lowest prevalence, at 0.5% [1]. In contrast to the United States Preventive Services Task Force (USPSTF) recommendations for universal HCV screening of all asymptomatic adults aged 18 to 79 years, countries with limited resources to HCV screening are at increased risk of sequelae from undiagnosed CHC due to the delays in diagnosis and receiving care. Additionally, data from the Centers for Disease Control and Prevention (CDC), which has tracked the incidence of acute HCV in the US since 1982, show that while acute infections in the US have decreased from the peak in 1989 to a nadir in 2010, there has been a disturbing trend of increasing cases since 2010 [1]. For example, the CDC estimates there has been a 4-fold increase in cases from 2010 to 2017: from roughly 11,800 cases of acute HCV in 2010 to 44,700 cases in 2017 [1]. The increase in cases has been linked to an ongoing opioid epidemic in the US, as persons who inject drugs (PWID) were found to have a much higher incidence of HCV infection, especially young adults [4,5]. The increased incidence and prevalence among PWID is not limited to the US, as shown in a systemic review that estimated that 52.3% of PWID have been exposed to HCV based on anti-HCV antibodies [6]. Data on the prevalence of HCV by HCV RNA was lacking in most countries, but of the countries that provided data, the lowest prevalence was noted in sub-Saharan Africa among PWID, and this was still listed at roughly 21.8% [6]. As more developing countries report data on PWID, it is apparent that this is a very high-risk group for CHC around the world.

The treatment of CHC has changed drastically within the last decade with the advent of DAAs, leading to a significant reduction in the use of ribavirin and an almost complete discontinuation of interferon. The vast majority of patients are treated with the newest DAA regimens, which are well tolerated and allow patients to achieve sustained virologic response (SVR) in excess of 98% [7]. Although management of CHC has become easier than ever before, there are still several considerations that highlight the utility of HCV prevention. For instance, increased usage of DAA therapy can lead to the development of viral resistance to current regimens, especially as resistance to treatment regimens has been documented during clinical trials [8]. Furthermore, liver disease can progress in a subset of patients, and some patients are still at risk for hepatocellular carcinoma (HCC) even with cure of CHC [9,10,11,12]. In addition, the specter of reinfection continues to follow those patients who are still deemed at high-risk for HCV infections, such as PWID, as treatment with DAAs has not been shown to give lasting immunity to HCV [13,14]. Access to treatment is another barrier to curative therapy [15]; several reports attest to the inability of patients to get treatment covered by public or private insurances in the US [15,16].

Given the current clinical burden of HCV infections, the World Health Organization’s (WHO) goal of a 90% reduction of HCV infections by 2030 is in jeopardy of not being achieved [1]. Therefore prevention, via vaccination, would be an ideal alternative to treatment in order to achieve this goal. Indeed, mathematical models have shown that a vaccine with even as little as 30% efficacy would still reduce the clinical burden of disease significantly [17,18,19,20].

## 3. Hepatitis C Virology and Immunology

### 3.1. Hepatitis C Virus Particle Composition

HCV is a positive-sense, enveloped, single-stranded RNA virus that is part of the genus Hepacivirus in the Flaviviridae family [21,22]. The viral genome is roughly 9 kb long and encodes a single polyprotein that is cleaved into multiple functional segments by viral and cellular proteases [22]. The functional segments can be further divided into structural and nonstructural (NS) components [22,23]. While many of the functions of these components are known, the full understanding is still incomplete [23].

HCV contains three structural proteins (core, E1, E2) that are located in the lipid envelope that covers the virus [22,24]. The structural proteins’ main functions are to facilitate assembly of the capsid structure and entry into cells [25,26]. The NS proteins, of which there are seven (p7, NS2, NS3, NS4A, NS4B, NS5A, and NS5B), are the main targets for current DAA therapies and perform a variety of functions that allow the virus to replicate within host cells [7,22,23].

### 3.2. Hepatitis C Virus Genome

The HCV genome is roughly 9 kb long with seven known genotypes, and there are at least 67 subtypes of HCV identified [27]. Given this genetic diversity, producing a vaccine that is active against all genotypes and subtypes is a monumental task. Moreover, the HCV NS5B polymerase, the target of drugs such as sofosbuvir, can generate genetically distinct but related species within a single host known as ‘quasispecies’ [28,29]. This is largely due to the fact that NS5B does not have a proof-reading mechanism to correct errors in replication [2,24]. This generation of quasispecies within an individual can select for viral resistance to host immune responses, further hindering development of a broadly reactive vaccine [29,30,31].

### 3.3. Hepatitis C Virus Immunology

The mechanism for how HCV is spontaneously cleared from the body has not been fully elucidated. Early research in chimpanzees has shown that neutralizing antibodies (nAb) against glycoprotein E2 was associated with viral clearance [32]. Multiple studies demonstrated that rapid development of nAb during the early phases of native infection in humans was also associated with increased ability to clear the virus [33,34].

While nAb are important in protecting against acute infection, cell-mediated immunity (CMI) also appears to play a role. Multiple studies have shown that depletion of both CD4 and CD8 T cells are linked to persistent infection in chimpanzees [35,36]. Studies in humans also showed that acute HCV infection led to a strong T-cell response [37]. A strong and persistent CD8 T-cell response was consistently observed in those people who achieved spontaneous resolution [37]. Based on these studies, it is clear that a vaccine that can elicit both an nAb and a CMI response would be ideal.

## 4. Limitations of Models for Hepatitis C Virus Vaccination

The development of an HCV vaccination has proven to be a difficult task not only due to complex HCV virology that makes identifying a universal target for antibodies elusive, but also because of limited appropriate preclinical animal models. While humans are the natural reservoir of HCV, human and even chimpanzee experimental conditions for a vaccine are ethically challenging. Many animal studies use chimeric humanized mouse models and HCV analogues from the Hepacivirus genus, which are attempted simulations of the actual human-HCV relationship [38].

## 5. Prior Hepatitis C Virus Vaccination Studies

### 5.1. Animal Models

Chimpanzee models were the first used to study the effect of HCV in animals and have played a critical role in understanding the immune response in the development of an HCV vaccine. A large variety of candidate HCV vaccines targeting humoral and/or cellular immunity exist: virus-like particles, recombinant protein, recombinant protein and peptide, DNA and viral vector, and recombinant protein-based strategies. Some of these also focused on envelope proteins and NS proteins.

Two studies from the chimpanzee model have progressed to human trials. A design using an antibody-based vaccine consisting of recombinant E1 and E2 glycoproteins from a genotype 1a virus adjuvanted with MF59 (immunological adjuvant) showed a strong antibody response and delayed onset of viremia, but it failed to provide sterilizing immunity [39]. A T-cell-based vaccine developed by Glaxo Smith Kline consists of a recombinant chimpanzee adenovirus (serotype 3) prime and modified vaccinia Ankara (MVA) boost (to increase immunogenicity) encoding the NS3-5B proteins from a genotype 1b virus [40]. This heterologous challenge resulted in a rapid recall of memory T-cell response with suppression of acute-phase viremia [41].

Another notable chimpanzee study used a recombinant protein vaccine against genotype 1a E1 and E2 in 21 chimpanzees and resulted in five becoming aviremic, resolution in 14, and chronic infection in two. The study demonstrated protection against acute and chronic infection [42,43]. A study using the same type of vaccine with antigen genotype 1b E1 or E24HVR-1 in four chimpanzees resulted in resolution in two and chronic infection in two [44]. The study showed that the E2 vaccine target was protected against chronic infection as opposed to the E1 target [44]. A study with a similar vaccine type and antigen GT1a E1E2 RIBIs in one chimpanzee resulted in CHC. Vaccination had delayed infection by 3 weeks but did not prevent persistence [45]. A study using recombinant protein and peptide vaccines with antigen GT2 E1E2 and HVR-1 peptides in one chimpanzee showed resolution. It protects against CHC infection [46]. Another study with the same vaccine type using antigen DNA GT1a E2 in two chimpanzees had also protected against CHC infection [47]. A study using viral vector vaccine type, using antigen GT1b core, E1, E2, p7, NS2, and NS3 in four chimpanzees showed protection against CHC infection [48]. Using DNA and viral vector type of vaccine with antigen GT1b core-E1E2, NS3 in four chimpanzees showed resolution in only one. The vaccine had reduced peak viremia but did not protect against CHC infection [49]. Study with antigen GT1b NS3-NS5B in five chimpanzees showed resolution in four and it protected against CHC infection in all except one [41]. Antigen GT1a NS3, NS5A, NS5B in one chimpanzee did suppress viremia early, but late resurgence and eventual persistence was noted [50]. Antigen GT1b core-E1E2 in six chimpanzees showed one being aviremic, resolution in one and chronic infection in four [51]. Antigen GT1a NS3-NS5B in two chimpanzees resulted in resolution in one and CHC infection in the other. The vaccine did suppress viremia in early stages [52]. Using vaccine type DNA and recombinant protein against antigen GT1a core, NS3; GT1b core, E1E2, NS3 in two chimpanzees showed reduction in peak viremia [53]. Using virus-like particles against GT1b core-E1E2 in four chimpanzees showed protection against chronic infection [54]. Eight vaccine studies in mice were notable: three of them were DNA vaccines. The first one used HCV strain GT1b, with targets E1, and E2 resulted in antibody responses toward GT1a, 1b, 2a, 2b, 3a, 4a, 5, 6, and a CD4+ T-cell response was observed [55]. The second one used the HCV strain GT1a H77, against targets core, E1, E1, p7, NS2, and NS3, which resulted in non-neutralizing antibody response; but both CD4+ and CD8+ T-cell responses were observed [56]. The third study used the HCV strain GT1b and GT3a, against targets NS3, NS4, and NS5b elicited both CD4 and CD8 T-cell response. Of two studies using peptide vaccine, using HCV strains of GT1b J4 and GT4a ED43 had elicited both CD4 and CD8 T-cell response [57,58]. Of the three studies using virus-like particle vaccine, the first one was conducted in both mice and pigs, using target core, E1 andE2, using the HCV strain GT1a H77, GT1b BK, GT2a JFH1, and GT3a, resulted in homologous neutralizing antibody response [59]. The second one, against linear E1 and E2 epitopes, resulted in a heterologous antibody response toward GT1a, 1b, and 2a [60].

Two studies used a rat Hepacivirus infection model to evaluate the effectiveness of T-cell immunization in preventing RHV persistence. A simian adenovirus vector vaccine strategy was effective at inducing complete protective immunity in the rat RHV model [61]. Vaccination of rats with adenoviral vectors expressing the RHV NS3-5B proteins produced T-cell responses with reduction in incidence of CHC infection. Inclusion of the E1 and E2 glycoproteins as vaccine targets resulted in increased protection from viral persistence [61,62].

### 5.2. Human Models

Given the promising data in animal models, several clinical trials involving humans have been performed [63,64,65,66]. These clinical studies have used varying vaccine types, including glycoproteins and viral vectors.

#### 5.2.1. Glycoprotein Vaccines in Human Models

Some Phase 1 trials have examined the immunogenicity of an HCV recombinant E1 and E2 glycoprotein as a candidate vaccine [63,64,65,66]. Frey constructed a randomized, double-blind, placebo-controlled, dose-escalation study over four vaccine doses with HCV E1/E2 in 60 healthy adults [63]. This study demonstrated lymphocyte proliferation responses to E1/E2 in participants with no significant difference in adverse effects across groups. The vaccine was well tolerated overall while stimulating a significant humoral and cell-mediated immune response that was interestingly not dose-dependent. Law also immunized patients with the recombinant glycoproteins E1E2 derived from a single HCV strain (genotype 1a) [64]. This study used 16 different vaccines, where three of the vaccines showed broad cross-neutralizing responses and at least one vaccine was pan-genotypic with responses against all HCV genotypes. While only a minority of the vaccines tested in this trial produced sufficient cross-neutralizing antibodies, it demonstrated the potential that a vaccine derived from a single strain of HCV could be used across other genotypes despite the differences in the structure of the E1/E2 glycoproteins [64]. Leroux used a 3:4 dose schedule in 20 male volunteers to elicit a cellular immune response, including a clear T helper type 1 response in all but one of their participants [65]. Similarly, Drane et al.’s study comprised 30 participants receiving three doses of a vaccine that produced antibody responses in all but one of the participants; however, this was limited by detectable CD8+ T-cell responses being only present in two of the eight participants receiving the highest dose of the vaccine [66].

#### 5.2.2. Viral Vector Vaccines in Human Models

Other human Phase 1–2 clinical trials have attempted to employ viral vectors to generate an immune response to viral HCV [67,68,69,70,71]; GT1b NS3-NS5B has been of particular interest. Most recently in a Phase 1–2 randomized, double-blind, placebo-controlled trial, Page used a recombinant chimpanzee adenovirus 3 vector priming vaccination (ChAd3-NSmut) followed by a recombinant modified vaccinia Ankara boost (MVA-NSmut) in 68 participants to produce HCV-specific T-cell responses [67]. While the study did elicit T-cell responses against HCV proteins, it did not ultimately prevent CHC infection as the incidence of CHC infection was not lower when compared to the placebo group. This phenomenon was postulated to be due to lower vaccine immunogenicity innately in the study population of persons who inject drugs. Meanwhile, the vector encoding the NS3, NS4, NS5A, and NS5B proteins of HCV genotype 1B in Swadling’s study showed a durable, sustained T-cell response in both CD4+ and CD8+ cells [68]. While this induction of T cells was not necessarily defined to convey protection of HCV in the study, it highlighted viral vectors as a potential modality in the development of a prophylactic HCV vaccine. Barnes in a Phase 1 trial used two rare serotype adenoviral vectors to prime T-cells to respond to multiple HCV strains consistent with protective immunity [69]. This approach proved to be well tolerated and highly immunogenic with CD4+ and CD8+ T-cell responses to a wide range of antigens that were detectible 1 year out. Hartnell extrapolated the need for HCV vaccination to also HIV-1 vaccination by developing a vaccination regimen involving replication-defective and serologically distinct chimpanzee adenovirus (ChAd3, ChAd63) vector priming followed by MVA boosts for simultaneous delivery of HCV NS and HIV-1 conserved (HIVconsv) region immunogens [70]. These co-administered vaccines were safely administered without reducing the immunogenicity of either vaccine. Esposito devised MHC class II invariant chain-adjuvanted viral vectored vaccines for humans in a dose-escalation study to enhance HCV-specific T-cell induction [71]. Overall, this T-cell immunity could play a critical role in HCV control and ideally improve vaccine efficacy.

### 5.3. Future Directions

The development of a broadly reactive HCV vaccine has progressed significantly in the last decade with multiple trials in both animal and human models. Unfortunately, there are still significant limitations in vaccine development as noted by the fact that all the above studies are still in early phases. An effective vaccine will likely need to integrate both an antibody response as well as a robust T-cell response, but to do this, a better understanding of the underlying mechanisms of how immune cells mediate short- and long-term protection is necessary. In addition, technological advancements—including usage of computational strategies such as computer-generated HCV virus vaccine sequences to elicit a cross-reactive T-cell response, which has been shown to be effective [72]—have not yet reached widespread use or study. Testing effective vaccines remains a challenge, and some experts have suggested that taking a small group of human volunteers to design a human infection model might be a useful and necessary next step [73]. Other experts have also noted that the overall public image of HCV is that it affects marginalized communities only, which has led to decreased interest, as evidenced by lack of funding. One Lancet article noted that in 2019 there were 39 human clinical trials for HIV vaccines whereas there were only 2 human clinical trials for HCV [74]. This suggests that more public education on the widespread nature of HCV and its implication to public health is also necessary to more quickly allow the science to progress. While many obstacles remain, the current research offers a hint on how to approach building a vaccine for HCV to augment the other available treatment strategies.

## Data Availability

Not applicable.
